# Statement of Retraction

**DOI:** 10.1080/10717544.2022.2157535

**Published:** 2023-01-18

We, the Editors and Publisher of the journal *Drug Delivery* have retracted the following article:

Lianlian Cao, Guojing Shao, Fengmei Ren, Minghua Yang, Yan Nie, Qian Peng & Peng Zhang (2021) Cerium oxide nanoparticle-loaded polyvinyl alcohol nanogels delivery for wound healing care systems on surgery, Drug Delivery, 28:1, 390-399, DOI: 10.1080/10717544.2020.1858998

Since publication, concerns have been raised regarding animal welfare, verifiable ethical approval, the mode of anesthesia/euthanasia, and whether any painkillers or antibiotics were given to animals post-injury.

The authors were contacted for an explanation but did not reply. As a result, we are retracting the article. The corresponding author listed in this publication has been informed.

We have been informed in our decision-making by our policy on publishing ethics and integrity and the COPE guidelines on retractions.

The retraction article will remain online to maintain the scholarly record, but it will be digitally watermarked on each page as ‘Retracted’.

